# Network-based regularization for high dimensional SNP data in the case–control study of Type 2 diabetes

**DOI:** 10.1186/s12863-017-0495-5

**Published:** 2017-05-16

**Authors:** Jie Ren, Tao He, Ye Li, Sai Liu, Yinhao Du, Yu Jiang, Cen Wu

**Affiliations:** 1 Department of Statistics, Kansas State University, 1116 Mid-Campus Drive N., 66506 Manhattan, KS USA; 2 Department of Mathematics, San Francisco State University, San Francisco, CA USA; 3 Department of Biostatistics, Yale University, New Haven, CT USA; 4 Division of Nephrology, School of Medicine, Stanford University, Palo Alto, CA USA; 5 Division of Epidemiology, Biostatistics, and Environmental Health, School of Public Health, University of Memphis, Memphis, TN USA

**Keywords:** Case–control association study, Network-based regularization, Regularized logistic regression, Type 2 diabetes, Variable selection

## Abstract

**Background:**

Over the past decades, the prevalence of type 2 diabetes mellitus (T2D) has been steadily increasing around the world. Despite large efforts devoted to better understand the genetic basis of the disease, the identified susceptibility loci can only account for a small portion of the T2D heritability. Some of the existing approaches proposed for the high dimensional genetic data from the T2D case–control study are limited by analyzing a few number of SNPs at a time from a large pool of SNPs, by ignoring the correlations among SNPs and by adopting inefficient selection techniques.

**Methods:**

We propose a network constrained regularization method to select important SNPs by taking the linkage disequilibrium into account. To accomodate the case control study, an iteratively reweighted least square algorithm has been developed within the coordinate descent framework where optimization of the regularized logistic loss function is performed with respect to one parameter at a time and iteratively cycle through all the parameters until convergence.

**Results:**

In this article, a novel approach is developed to identify important SNPs more effectively through incorporating the interconnections among them in the regularized selection. A coordinate descent based iteratively reweighed least squares (IRLS) algorithm has been proposed.

**Conclusions:**

Both the simulation study and the analysis of the Nurses’s Health Study, a case–control study of type 2 diabetes data with high dimensional SNP measurements, demonstrate the advantage of the network based approach over the competing alternatives.

## Background

Type 2 diabetes mellitus (T2D), a chronic metabolic disorder, has been a major public health concern for years. An estimated 366 million cases of T2D over the world are expected by the year 2030 [1]. To better understand T2D etiology, significant efforts have been devoted to the identification of genetic markers that may contribute to the predisposition of the disease. The large scale genome–wide association studies (GWAS) has proven to be powerful in finding the association between individual genetic variant (like SNPs) and complex diseases, including type 2 diabetes. However, those identified SNPs from existing studies can only account for about 10% of the genetic variance of type 2 diabetes [2], which motivate the development of more advanced statistical methodologies with the hope to explain the missing heritability.

One major limitation shared by many of the previous studies, especially the early ones, is that they are marginal in the sense that one or a small number of genetic factors are analyzed at a time. Since complex disease phenotypes are associated with the joint effects of multiple genetic factors, signals with weak or moderate marginal but strong joint effects may not be captured by the marginal analysis.

As unprecedented amount of high dimensional omics data has been generated from high–throughput profiling studies, extensive regularized variable selection methods such as LASSO [3] and elastic net [4], have been proposed to identify genes that are associated with disease phenotypes, with the genes being treated as variables. More recently, to incorporate the interconnection information, or network structure existing among genetic variants into the selection procesure, the network–constrained regularization approaches have been developed, as in Li and Li [5] and Huang et al. [6], among many others. In particular, Huang et al. [6] developed the sparse Laplacian shrinkage (SLS) penalty built upon the combination of MCP (Zhang [7]) and Laplacian quadratic associated with a graph. They also demonstrated that in high dimension settings with *p* ≫ *n* under reasonable assumptions, SLS is selection consistent and equivalent to the oracle Laplacian shrinkage estimator with high probability.

This study has been partially motivated by analyzing the case control data from the Nurses’s Health Studies (NHS) and studies alike. As a major component of the Gene Environment Association Studies Initiative, NHS was launched in 1976 in order to identify important genetic variants related to type 2 diabetes and gene–trait association under environmental exposures [8]. To accommodate the linkage disequilibrium (LD) existing among SNPs, we adopt a network measure and incorporate it in SLS. We further extend the SLS into the penalized logistic regression model for the analysis of the T2D case control data, and develop an efficient coordinate descent based algorithm. Compared with the alternatives, the proposed method can borrow strength from the correlation among SNPs and leads to more meaningful identification of important ones.

We first introduce the data and model settings, and describe the proposed approach. An efficient computational algorithm is subsequently developed. Simulation study demonstrates the significant advantage of the proposed approach over multiple competing alternatives. We analyze NHS type 2 diabetes data with high dimensional SNP measurements.

## Methods

Denote the *i* ^th^ subject by using the subscript $i$. Let (*X*_*i*_, *Y*_*i*_), *i* = 1, …, *n* be *n* independent and identically distributed random vectors. *Y*_*i*_ is the binary response variable where *y*_*i*_ = 1 indicating the case of disease, and 0 otherwise. *X*_*i*_ is the *p*–dimensional design vector of SNPs. Assuming that *y*_*i*_ follows a binomial distribution, then

$$ P\left({y}_i=1\Big|{\eta}_i\right)={\pi}_i=\frac{e^{\eta_i}}{1+{e}^{\eta_i}} $$

where *η*_*i*_ is the *i* ^th^ component of *η* = *Xβ*, and *β* = (*β*_*i*_, …, *β*_*p*_)^*T*^ is the regression coefficient vector. The corresponding loss function is the negative log-likelihood

1 $$ L\left(\eta \right)=\frac{1}{n}{\displaystyle \sum_{i=1}^n{L}_i\left({\eta}_i\right)=-\frac{1}{n}{\displaystyle \sum_{i=1}^n \log P\left({Y}_i={y}_i\Big|{\eta}_i\right)}} $$

## Regularized logistic regression

As the T2D disease status is only affected by a small number of important SNPs that are associated with the disease, and the dimensionality of the total number of SNPs is much larger than the sample size *n*, the problem is of a “large *p*, small *n*” nature. Regularization is a natural tool for such type of problem appropriate in both biological and statistical sense. By imposing penalty function to the loss function in (1), we have the following penalized likelihood

2 $$ Q\left(\beta \right)=-\frac{1}{n}{\displaystyle \sum_{i=1}^n\left\{{y}_i \log {\pi}_i+\left(1-{y}_i\right) \log \left(1-{\pi}_i\right)\left\}+ P\right(\beta; \lambda, \gamma \right)} $$

where *P*(*β*; *λ*, *γ*) is the penalty function with tuning parameters *λ* and *γ*. A seemingly straightforward choice for the penalty is

$$ P\left(\beta; \lambda, \gamma \right)={\displaystyle \sum_{m=1}^p\rho \left({\beta}_m;{\lambda}_1,\gamma \right)} $$

where $\rho \left( t;{\lambda}_1,\gamma \right)={\lambda}_1{\displaystyle {\int}_0^{\left| t\right|}\Big(1-\frac{x}{\gamma {\lambda}_1}}\Big){}_{+} d x$ is the MCP penalty with tuning parameter *λ*_1_ and regularization parameter γ (Zhang [7]).

For the SNPs, MCP is imposed on their regression coefficients. Penalized regression will shrink some components of coefficient vector *β* to zero, which indicates that the corresponding SNPs are not associated with the disease status *y*. SNPs with nonzero coefficients are treated as important variants. A major limitation of MCP here is that it ignores the interconnections among SNPs, while the high correlation among genetic variants, including SNPs, have been widely observed and reported due to LD. We use a network structure to describe the correlation pattern among SNPs. In a SNP network, a node corresponds to a SNP, and if the two SNPs are statistically or biologically associated, the two corresponding nodes are connected. To incorporate the network information, we adopt the sparse Laplacian penalty from Huang et al. [6] as follows:

3 $$ P\left(\beta; \lambda, \gamma \right)={\displaystyle \sum_{m=1}^p\rho \left({\beta}_m;{\lambda}_1,,\gamma \right)}+{\lambda}_2{\displaystyle \sum_{1\le m< k\le p}\left|{a}_{m k}\right|}{\left[{\beta}_m-\operatorname{sgn}\left({a}_{m k}\right){\beta}_k\right]}^2 $$

where |*a*_*mk*_| is the measure of connection intensity between SNP *x*_*m*_ and *x*_*k*_. The first term of (3) is a summation of MCPs, promoting sparsity in the estimated model. The role of the second term is to encourage smoothness among the coefficient profiles of the related SNPs. Furthermore, the second term can be associated with a Laplacian matrix for a properly defined undirected weighted graph corresponding to the SNPs. As shown in Huang et al. [6], the penalty in (3) is capable of taking correlation structure into account without introducing extra bias, consequently it outperforms a large class of network–constrained penalty functions. The oracle property has also been rigorously established. Therefore, we choose (3) and extend it to the penalized logistic regression model for the analysis of case control type 2 diabetes data.

The network adjacency measure, |*a*_*mk*_|, is perhaps the most crucial characteristic in a network to quantify strength of connection between any two nodes (Zhang and Horvath [9]). Denote *A* = (*a*_*mk*_, 1 ≤ *m*, *k* ≤ *p*) as the adjacency matrix, and let *r*_*mk*_ be the corresponding Pearson correlation coefficient. We propose to use *a*_*mk*_ = *r*_*mk*_^*α*^ ⋅ *I*{ | *r*_*mk*_| > *r*_*c*_} with *α* =5. This measure keeps the strong correlations while downweighing the weak ones. In addition, it guarantees that *a*_*mk*_ and *r*_*mk*_ have the same sign. Compared with the threshold *r*_*c*_ which determines whether the edge joins the corresponding nodes in a network, the power only denotes the relative strength of connection, and does not influence the network structure. Thus *α* can be chosen via an ad hoc fashion. The correlation cutoff *r*_*c*_ is calculated based on the Fisher transformation *z*_*mk*_ = 0.5 log((1 + *r*_*mk*_)/(1 − *r*_*mk*_)). If the correlation between *m* ^th^ and *k* ^th^ predictor is zero, then $\sqrt{n-3}\ {z}_{mk}$ approximately follows a standard normal distribution *N*(0,1), which can be used to determine a threshold *c* for $\sqrt{n-3}\ {z}_{mk}$. Subsequently, the corresponding threshold for *r*_*mk*_ is ${r}_c=\frac{ \exp \left(2 c/\sqrt{n-3}\right)-1}{ \exp \left(2 c/\sqrt{n-3}\right)+1}$. Such a network is weighted and sparse. We acknowledge that there are other ways of constructing the network adjacency matrix, and conjecture that they are equally applicable. Since our main purpose is not to compare the constructions of different networks, we focus on this particular network structure in this paper.

## Computation

Huang et al. [6] adopted a coordinate descent algorithm to obtain the sparse Laplacian shrinkage estimate when the continuous response variable follows a normal distribution. However, this cannot be applied to a binary response directly. We develop a coordinate descent based iteratively reweighed least squares (IRLS) algorithm for the logistic regression, which yields a form the same as the quadratic approximation to the penalized objective function based on Taylor expansion about current estimates. Denote *β* ^(*d*)^ as the value of the regression coefficients at the beginning of the *d*th iteration, the quadratic approximation to (2) is

$$ Q\left(\beta \right)\approx -\frac{1}{2 n}{\left(\tilde{y}- X\beta \right)}^T W\left(\tilde{y}- X\beta \right)+ P\left(\beta;\ \lambda,\ \gamma \right) $$

where *W* is an *n* × *n* diagonal matrix of weights with elements *w*_*i*_ = *π*_*i*_(1 − *π*_*i*_), and $\tilde{y}$ is the working response, defined as

$$ \tilde{y}= X{\beta}^{(d)}+{W}^{-1}\left( y-\pi \right) $$

where *π* = (*π*_1_, …, *π*_*n*_)^*T*^ is evaluated at current parameters *β* ^(*d*)^. The residuals after each iteration can be expressed as

$$ r=\tilde{y}- X{\beta}^{(d)}={W}^{-1}\left( y-\pi \right) $$

Let *v*_*m*_ = *n* ^− 1^ *X*_*m*_^*T*^ *WX*_*m*_. For a standardized design matrix *X*, we can have

$$ \begin{array}{c}{z}_m={n}^{-1}{X}_m^T W\left(\tilde{y}-{X}_{- m}{\beta}_{- m}\right)\\ {}={v}_m\left({n}^{-1}{X}_m^T r+{\beta}_m^{(d)}\right)\end{array} $$

Here, *v*_*m*_ needs to be re-weighted in every iteration, leading to increased computational cost. As the Hessian terms can be approximated by an exact upper bound (Krishnapuram et al. [10]), we can set *w*_*i*_ all equal to $\frac{1}{4}$. Define *u*_*m*_ and *t*_*m*_ at iteration *d* as

4 $$ \begin{array}{c}{u}_m={z}_m+{\lambda}_2{\displaystyle \sum_{k= m+1}^p\left|{a}_{m k}\right|}{\beta}_k\\ {}{t}_m=\frac{1}{4}+{\lambda}_2{\displaystyle \sum_{k= m+1}^p\left|{a}_{m k}\right|}\end{array} $$

Then the close form update of *β* ^(*d* + 1)^ can be obtained as

5 $$ {\beta}_m^{\left( d+1\right)}=\left\{\begin{array}{l}\frac{S\left({u}_m,\ {\lambda}_1\right)}{t_m-1/\gamma}\kern1.25em \mathrm{if}\ \left|{u}_m\right|\ \le\ {t}_m\gamma {\lambda}_1\hfill \\ {}\frac{u_m}{t_m}\kern4em \mathrm{if}\ \left|{u}_m\right| > {t}_m\gamma {\lambda}_1\hfill \end{array}\right. $$

where *S*(⋅) is the soft thresholding function. With fixed tuning parameters *λ*_1_ and *λ*_2_, the coordinate descent algorithm proceeds as follows.
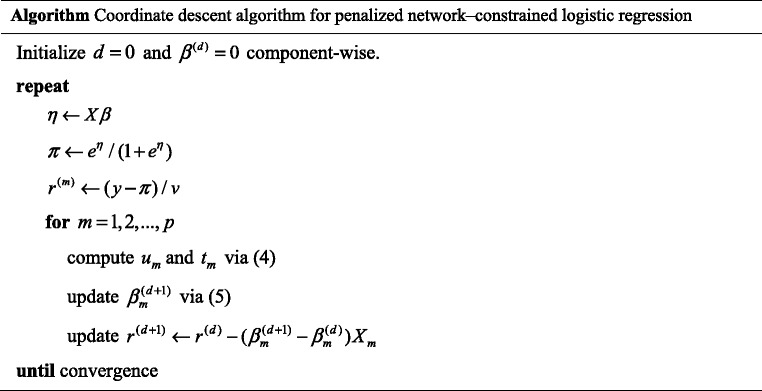


The convergence is achieved when the *L*_2_ difference between *β* estimates from two contiguous iterations is smaller than a predefined threshold. Tuning parameters *λ*_1_ and *λ*_2_ control the sparsity in SNP selection and smoothness among coefficient profiles, respectively. They can be chosen from cross validation based methods. We search over a two-dimensional discrete grid of values for *λ*_1_ and *λ*_2_, and select the optimal pair in terms of testing misclassification rate. In penalized logistic regression, regularization parameter γ needs to be larger than 1/*w*_*i*_ for MCP. We set it as 4.5 in the simulation study since it has been observed that smaller γ yield slightly better results.

## Results

### Simulation

We evaluate the performance of the proposed approach through extensive simulation studies. Both categorical and continuous predictors are considered, and they correspond to SNP and gene expression data, respectively. We first generate a *n* × *p* matrix of gene expressions, where *n* = 500 and *p* = 750, from a multivariate normal distribution. For the 750 genes, there are 100 clusters with 5 genes per cluster. The gene expressions have been marginally standardized. We consider two correlation structures. (1) the auto-regression (AR) structure, in which gene *i* and *j* within the same cluster have correlation coefficients *ρ* ^|*i* − *j*|^, and they are independent cluster–wisely. (2) the block structure, in which the within cluster correlation coefficient is *ρ*, and gene expressions in different clusters are independent. We consider *ρ* =0.1, 0.5 and 0.9 for both structures. In addition to the 500 by 750 matrix of gene expressions, a 1000 by 1500 matrix has also been generated with 150 clusters and 10 genes per cluster following the same correlation structures. The SNP data are simulated by dichotomizing expression values of each gene at the 1st and 3rd quartiles, with the 3–level (2,1,0) for genotypes (AA,Aa,aa) respectively. For both combinations of (*n*, *p*), (500, 750) and (1000, 1500), 10% of clusters are randomly selected to have nonzero regression coefficients, which are generated from *Unif* [0.25,0.75]. The binary response can subsequently be simulated. We choose the tuning parameters based upon the prediction performance of the corresponding model in an independently simulated validation dataset.

For comparison, we consider three alternative approaches, LASSO, elastic net and MCP. LASSO is perhaps so far the the most widely used penalization approach for the analysis of genomic data. In contrast to LASSO, elastic net encourages the grouping effects among genomic features. MCP is equivalent to the proposed approach when *λ*_2_ = 0 in (3). Comparison with MCP as well as elastic net will directly demonstrate the advantage of each penalty term in the formulation (3). For convenience, we term the network approach, MCP, elastic net and LASSO as A1, A2, A3 and A4, respectively.

**Fig. 1:**
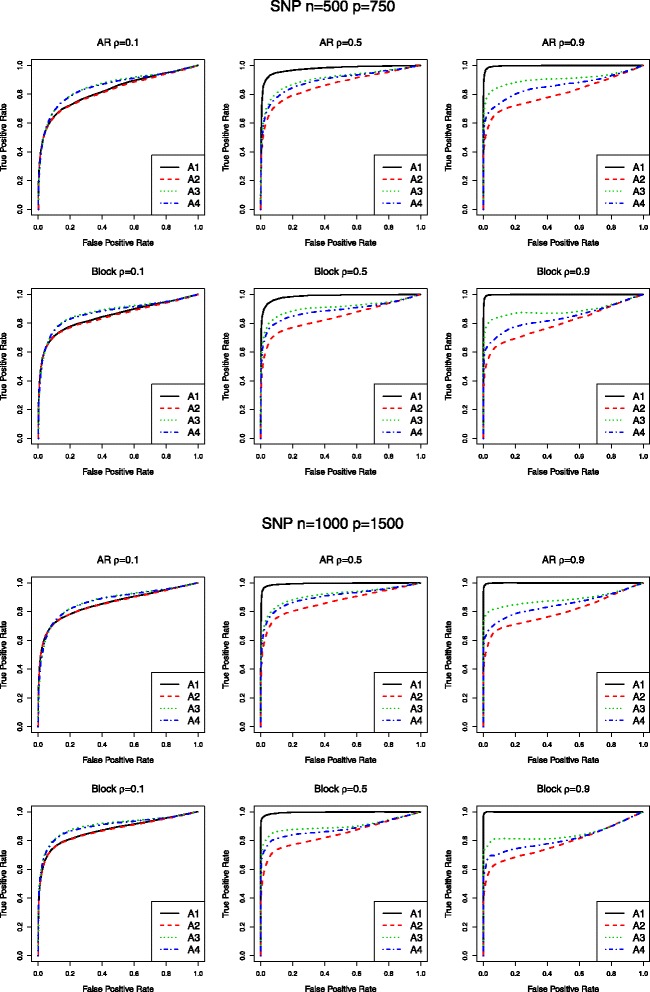
ROC curves for SNP data. ROC curves corresponding to Table 1

**Fig. 2:**
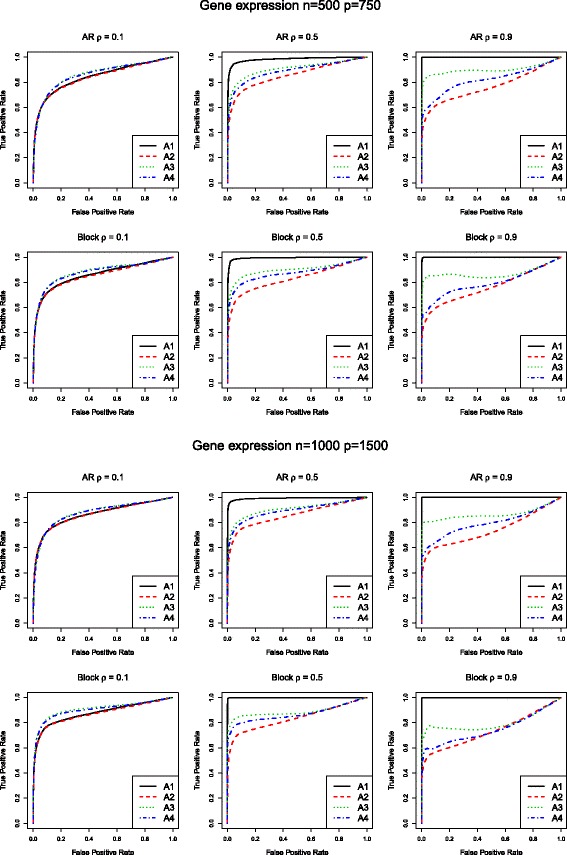
ROC curves for Gene Expression data. ROC curves corresponding to Table 2

**Fig. 3:**
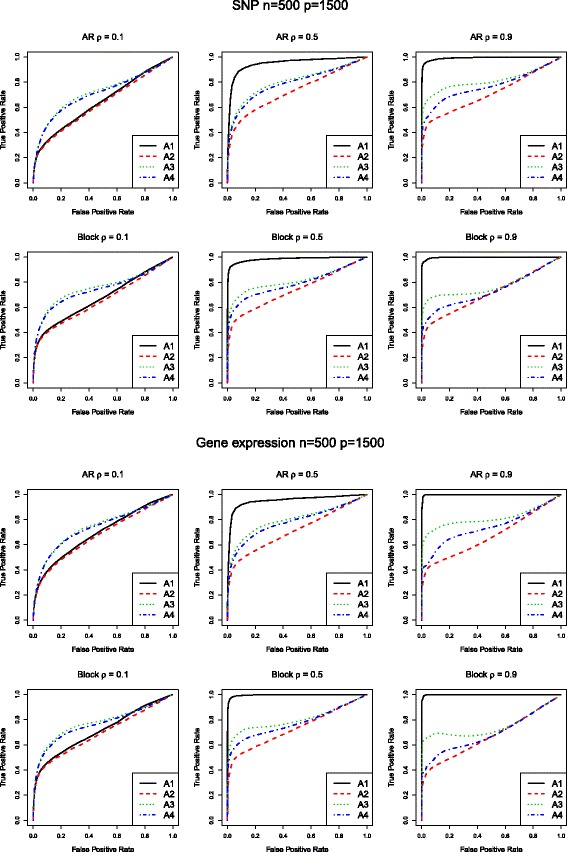
ROC curves for *(n, p)* = (500,1500). ROC curves corresponding to Table 3

Simulation results for the SNP data are tabulated in Table 1. We can observe that from the upper panel where (*n*, *p*) = (500, 750), A1 (network) and A2 (MCP) have similar performance when correlation is low. As correlation increases, the proposed one starts to outperform A2. For example, when *ρ* = 0.9 under AR correlation structure, A1 can identify most of the 75 true positives, 74.98 (sd 0.14), with a small number of false positives 9.74 (sd 13.31). A2 identifies similar number of false positives with a much lower number of true positives 31.22 (sd 4.57). Out of all the four approaches, A3 (elastic net) always has the largest false positives and A4 (LASSO) is inferior to A2 in general. Consistent patterns have been observed under other scenarios in Table 1. In addition, we examine the performances using the ROC curves. The ROC curves corresponding to Table 1 are given in Fig. 1, which clearly show that A1 outperforms A2–4. Additional simulation results for gene expression data are given in Table 2 and Fig. 2, which also demonstrate the merit of the network approach over the alternatives when moderate to strong correlation exists among genetic variants. To further examine the performance of the proposed approach, we also conduct simulation under *n* = 500 and *p* = 1500. Results are summarized in Table 3 and Fig. 3. Both the identification accuracy in Table 3 and ROC curves in Fig. 3 demonstrate the superiority of the proposed A1 over alternatives.

**Table 1:** Simulation for SNP data: mean(sd) of true positives (TP) and false positives (FP) based on 100 replicates. Upper panel: (*n*, *p*) = (500, 750); Lower panel: (*n*, *p*) = (1000, 1500)

		A1	A2	A3	A4
AR *ρ* = 0.1	TP FP	41.04(7.07) 37.02(28.65)	39.28(7.40) 32.02(27.49)	61.14(2.86) 169.38(18.79)	53.30(4.23) 83.58(21.61)
AR *ρ* = 0.5	TP FP	64.86(6.89) 32.12(30.54)	41.46(8.62) 21.84(24.23)	64.66(2.89) 136.22(11.93)	57.22(3.96) 60.10(11.60)
AR *ρ* = 0.9	TP FP	74.98(0.14) 9.74(13.31)	31.22(4.57) 4.30(3.90)	64.18(2.68) 88.84(12.52)	51.98(3.11) 30.52(9.67)
Block *ρ* = 0.1	TP FP	47.70(8.62) 61.40(77.21)	45.82(8.92) 52.22(71.40)	62.70(2.75) 162.66(18.66)	55.70(4.20) 79.52(19.80)
Block *ρ* = 0.5	TP FP	67.92(6.35) 31.40(22.18)	39.62(7.05) 15.74(13.65)	65.30(3.23) 116.16(13.10)	57.00(3.68) 43.96(13.36)
Block *ρ* = 0.9	TP FP	72.06(4.16) 12.22(16.30)	30.28(6.08) 5.08(6.22)	64.08(2.29) 83.30(12.76)	50.94(3.44) 27.86(10.81)
AR *ρ* = 0.1	TP FP	94.20(12.15) 77.10(50.90)	94.16(12.28) 77.62(51.35)	126.22(4.55) 317.70(22.91)	113.78(6.27) 165.26(31.10)
AR *ρ* = 0.5	TP FP	142.68(3.61) 28.12(25.50)	84.28(10.71) 30.82(26.16)	130.12(3.79) 233.08(16.09)	116.66(4.48) 103.42(20.82)
AR *ρ* = 0.9	TP FP	149.96(0.20) 33.86(33.25)	64.40(9.54) 9.26(9.25)	124.06(3.05) 105.46(17.51)	98.96(4.46) 25.74(9.43)
Block *ρ* = 0.1	TP FP	101.22(12.37) 72.18(45.32)	98.96(13.55) 67.76(46.36)	132.46(3.82) 274.58(18.19)	121.84(4.57) 129.70(19.58)
Block *ρ* = 0.5	TP FP	145.68(5.93) 62.78(58.00)	75.84(9.18) 16.22(13.47)	129.94(3.13) 144.26(17.69)	114.92(4.25) 45.00(12.74)
Block *ρ* = 0.9	TP FP	147.40(8.42) 27.56(30.66)	56.32(9.93) 6.36(7.59)	120.78(4.10) 81.14(13.58)	92.62(6.34) 19.60(9.02)

**Table 2:** Simulation for Gene expression data: mean(sd) of true positives (TP) and false positives (FP) based on 100 replicates. Upper panel: (n, p) = (500, 750); Lower panel: (n, p) = (1000,1500)

		A1	A2	A3	A4
AR *ρ* = 0.1	TP FP	43.50(8.64) 49.50(45.54)	40.48(8.48) 35.58(32.92)	61.46(2.92) 163.08(15.56)	53.50(4.59) 76.92(19.67)
AR *ρ* = 0.5	TP FP	68.74(9.23) 29.64(25.13)	38.56(7.04) 17.06(15.23)	64.46(2.56) 127.36(17.11)	55.54(3.69) 54.94(16.08)
AR *ρ* = 0.9	TP FP	74.34(2.00) 10.48(13.50)	27.68(5.58) 3.50(3.72)	65.30(1.62) 76.82(14.10)	45.50(3.11) 23.80(9.96)
Block *ρ* = 0.1	TP FP	44.92(8.75) 40.58(40.79)	42.92(7.96) 30.84(24.45)	64.20(2.91) 161.44(13.32)	56.82(3.70) 77.24(17.99)
Block *ρ* = 0.5	TP FP	72.72(4.01) 22.08(30.01)	38.94(6.86) 15.06(18.84)	65.36(2.84) 107.18(12.58)	56.88(3.54) 38.70(11.31)
Block *ρ* = 0.9	TP FP	70.12(4.29) 5.88(10.48)	25.24(4.38) 1.92(1.52)	64.62(2.58) 75.42(11.49)	43.50(3.22) 23.16(7.77)
AR *ρ* = 0.1	TP FP	88.86(15.09) 69.58(56.10)	86.72(15.32) 61.56(49.57)	126.16(4.51) 312.28(24.32)	113.38(5.89) 159.98(27.91)
AR *ρ* = 0.5	TP FP	146.14(2.65) 43.62(37.20)	81.68(12.44) 24.20(20.15)	129.88(3.27) 217.86(17.82)	115.14(4.93) 93.60(16.22)
AR *ρ* = 0.9	TP FP	149.42(2.26) 27.98(37.43)	52.64(7.78) 4.22(5.28)	122.50(4.03) 97.06(14.09)	82.42(5.24) 23.26(8.58)
Block *ρ* = 0.1	TP FP	91.70(12.04) 47.36(31.01)	88.92(12.70) 41.96(28.95)	131.92(3.26) 264.58(20.20)	120.76(4.32) 124.84(17.73)
Block *ρ* = 0.5	TP FP	148.38(4.95) 24.78(29.56)	74.02(10.59) 17.46(12.93)	127.70(3.70) 127.80(17.06)	110.94(4.60) 37.10(12.48)
Block *ρ* = 0.9	TP FP	145.10(4.85) 18.54(32.97)	45.60(7.37) 2.74(2.47)	117.90(3.71) 69.06(12.25)	73.56(4.73) 14.06(5.94)

**Table 3:** Simulation for (*n*, *p*) = (500, 1500): mean(sd) of true positives (TP) and false positives (FP) based on 100 replicates. Upper panel: SNP data; Lower panel: gene expression data

		A1	A2	A3	A4
AR *ρ* = 0.1	TP FP	48.52(12.31) 78.29(41.61)	37.57(12.59) 64.29(51.34)	57.55(14.03) 95.11(36.75)	46.60(14.58) 63.32(30.63)
AR *ρ* = 0.5	TP FP	126.91(10.98) 78.69(33.05)	59.37(10.48) 54.19(29.45)	92.57(6.94) 139.60(27.36)	78.83(6.70) 87.37(17.78)
AR *ρ* = 0.9	TP FP	148.43(9.83) 61.36(48.01)	47.82(12.45) 21.41(60.62)	105.29(5.34) 135.23(20.22)	80.05(4.81) 80.03(12.34)
Block *ρ* = 0.1	TP FP	67.16(12.67) 91.44(40.23)	52.23(11.89) 63.03(42.99)	81.51(7.92) 124.56(28.34)	70.51(7.25) 82.16(19.66)
Block *ρ* = 0.5	TP FP	146.63(7.27) 81.27(63.42)	57.24(13.70) 32.85(42.69)	105.11(4.96) 143.77(19.63)	89.64(4.46) 87.04(12.76)
Block *ρ* = 0.9	TP FP	143.11(5.33) 28.85(41.83)	43.92(10.42) 11.99(19.14)	105.69(5.16) 133.15(20.07)	82.12(5.11) 75.53(12.27)
AR *ρ* = 0.1	TP FP	47.41(10.56) 80.08(38.04)	45.27(11.08) 73.56(39.11)	61.12(12.18) 100.51(34.81)	49.37(13.06) 64.09(28.80)
AR *ρ* = 0.5	TP FP	137.85(9.22) 74.79(30.71)	50.61(9.79) 31.28(14.74)	96.19(6.07) 142.99(25.91)	81.23(5.95) 91.92(16.12)
AR *ρ* = 0.9	TP FP	148.80(3.65) 40.33(31.15)	38.57(9.09) 7.09(10.13)	107.95(4.73) 129.96(17.41)	70.91(4.23) 77.48(11.09)
Block *ρ* = 0.1	TP FP	60.37(11.61) 83.04(48.75)	53.43(12.30) 60.64(41.84)	86.67(6.85) 133.73(26.74)	74.79(5.96) 88.28(18.60)
Block *ρ* = 0.5	TP FP	140.67(14.14) 73.60(66.06)	53.89(13.26) 27.35(31.89)	104.83(4.67) 138.88(17.85)	86.65(4.25) 82.63(13.30)
Block *ρ* = 0.9	TP FP	145.12(5.00) 19.43(23.30)	33.07(7.61) 3.79(6.54)	106.67(5.81) 123.37(19.61)	64.76(4.71) 67.60(9.78)

In addition to the identification results and the ROC curves, we acknowledge that plots of piece-wise solution path can be adopted to investigate the similarity and difference among different regularization methods, especially when the number of predictors (features) entering the model is moderate or small. In our simulation study, the number of important SNPs and gene expressions is large, therefore such an approach is not pursued.

### Real data analysis

As described in the background section, we analyze Nurses’ Health Study (NHS), a case control study of type 2 diabetes which are part of the Gene Environment Association Studies. Detailed information of the datasets are available from Hu et al. [11]. We focus on SNPs in several important pathways potentially related to T2D. They are the Wnt signaling pathway, cell cycle pathway and p53 signaling pathway. After cleaning the data through matching phenotypes and genotypes, removing SNPs with minor allele frequency (MAF) less than 0.05 or deviation from Hardy–Weinberg equilibrium, the working dataset contains 3391 subjects. There are 5079 SNPs in the Wnt signaling pathway, and 3793 SNPs in the cell cycle and p53 signaling pathway.

**Fig. 4:**
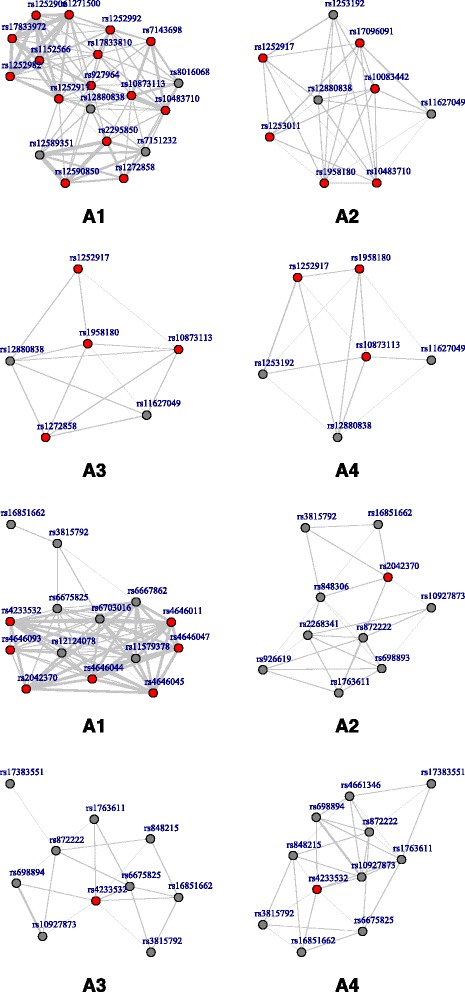
Subnetworks of DAAM1 (upper panel) and CASP9 (lower panel). SNPs connected in the network are joined with edges

**Fig. 5:**
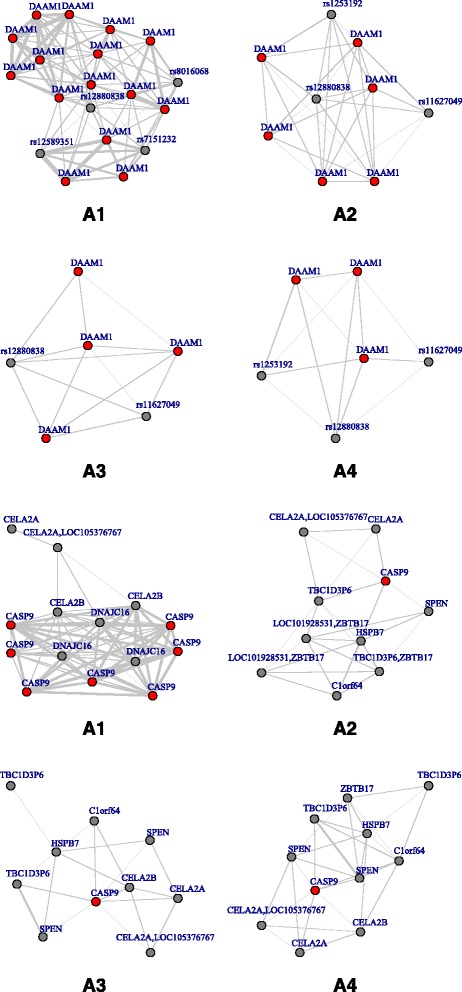
Subnetworks of DAAM1 (upper panel) and CASP9 (lower panel). Gene names are corresponding to the SNP id in Fig. 4

We first apply all the 4 methods on the Wnt signaling pathway. The A1–4 identify 834, 841,847 and 848 SNPs that are associated with T2D, respectively. As a representative example, we examine closely gene DAMM1 and its subnetwork. DAMM1 is reported to be associated with diabetic nephropathy, a common complication of type 2 diabetes (Sapienza et al. [12] and Kavanagh et al. [13]). The upper panel of Fig. 4 shows the subnetwork of DAMM1, where the red nodes indicate the SNPs from DAMM1. In the subnetworks, thickness of edges denotes the strength of correlation between SNPs. It can be clearly observed that the proposed approach has identified much more highly correlated SNPs, since the interconnections among SNPs have been accommodated by the approach that incorporates the network structure information. The network approach (A1) selects 19 SNPs and 15 belong to DAMM1, while other 3 approaches only identify 9,6 and 6 SNPs correspondingly. A1 leads to a more tightly connected network, which is consistent with our findings in the simulation study that it promotes the interconnections among SNPs. Furthermore, the proposed one identifies SNP rs1252906, which plays a crucial role in the progression of nephropathy (Kavanagh et al. [13]). Other methods fail to identify this important SNP. The genes corresponding to the SNPs in the subnetworks are given in the upper panel of Fig. 5.

The analysis has also been carried out on the SNPs combined from the cell cycle pathway and p53 signaling pathway. There are 814, 828, 827 and 829 SNPs identified by A1–4 correspondingly. We focus on the subnetwork of gene CASP9, which is one of the key players in inducing cell apoptosis (Cnop et al. [14]). Previous studies show that CASP9 is associated with diabetic retinopathy, a common and serious complication of type 2 diabetes (Baharian et al. [15] and Looker et al. [16]). In the NHS study, CASP9 has a total of 11 SNPs. The subnetwork of CASP9 is shown in the lower panel of Fig. 4. The proposed method identifies a subnetwork which has 7 SNPs from CASP9, and the other 7 SNPs from gene CELA2A, CELA2B and DNAJC16. Both CELA2A and CELA2B encode protein elastases, which hydrolyze elastin and many other proteins. DNAJC16 is a member of heat shock protein family (Hsp40). And it has been found in multiple studies that Hsp40 are related to cell apoptosis in type 2 diabetes (Laybutt et al. [17] and Chien et al. [18]. It is very interesting that CELA2A, CELA2B, CASP9 and DNAJC16 locate on chromosome 1 as a cluster. CELA2A is also identified by the rest 3 approaches, while CELA2B is only not in the subnetwork identified by A2(MCP). Overall, the network effects of CASP9, CELA2A, CELA2B and DANJC16 on type 2 diabetes, especially diabetic retinopathy, worth further investigations.

## Discussion

In this paper, we develop a network–based regularized logistic regression model for the analysis of high dimensional genetic data and identification of important SNPs in the case–control study of type 2 diabetes. Advancing from existing studies, the proposed one has desired property to take correlation pattern among genetic variants into account without incurring extra bias. We provide an efficient iteratively reweighed least squares (IRLS) algorithm within the coordinate descent framework. The computational cost has been significantly reduced due to convenient approximations to the original regularized log likelihood function. Simulation demonstrates that the proposed one outperforms closely related alternatives.

Computation feasibility is an important practical consideration for high–dimensional regularization methods. In simulation, the CPU time (in minutes) of applying the proposed method on 100 replicates of simulated SNP data with *n* = 1000, *p* = 1500 and AR structure is 218.3 on a regular laptop. In the case study, the CPU time for analyzing the Wnt pathway with *n* = 3391 and *p* = 5079 is 70.16. The proposed method can be potentially applied to larger datasets with a reasonable computation time. It has been widely acknowledged in Fan and Lv [19], Jiang et al. [20] and studies alike that regularization methods have to be coupled with screening strategy to accommodate ultra-high dimensional data from for instance, large-scale GWAS studies. The proposed network constrained regularization method can be implemented in such a two stage framework. Further investigations are intriguing but beyond the scope of this paper, and will be postponed to the future.

The methodological development in this article has been partially motivated by the analysis of the datasets from Nurese’s Health Study (NHS). In the past, this study has been extensively investigated under marginal methods ([21] and [22]) which ignores the joint effects of the SNPs. In addition, although studies like Wu, Cui and Ma [23] consider the effects of SNPs jointly within the penalization framework for continuous phenotypes, the correlation among the SNPs still have not been fully taken into account. The proposed approach quantifies the strength of correlation among SNPs via network structure and is able to incorporate the correlation in the identification of important SNPs. In the case study, we have identified interesting subnetworks with respect to genes closely related to T2D. In this work, we have focused on methodological development. More thorough bioinformatics and functional investigations will be needed in the future to fully understand the identified results.

## Conclusions

The network-constained logistic regulaization method proposed in this study has demonstrated superior performance in identifying important genetic variants from both simulation study and the Nurese’s Health Study, a case–control study of type 2 diabetes with high dimensional SNP measurements. The network term in the regularized loss function accomodates the LD widely present among SNPs, which guarantees the advantage of the developed one over the competing alternative methods.

## Abbreviations

AR: Auto-regression
GWAS: Genome–wide association studies
IRLS: Iteratively reweighed least squares
LASSO: Least Absolute Shrinkage and Selection Operator
LD: Linkage disequilibrium
MAF: Minor allele frequency
MCP: Minimax concave penalty
NHS: Nurses’s Health Studies
SLS: Sparse Laplacian shrinkage
T2D: Type 2 diabetes
